# Exercise and Brain Health in Postmenopausal Women: A Review of Cognitive Benefits, Mechanisms, and Neurodegeneration Prevention

**DOI:** 10.3390/neurosci7040090

**Published:** 2026-08-17

**Authors:** April Mae Flynn, Ahmed Hankir, Mahera Abdulrahman, Nouf Al-Rumaihi, Frederick Robert Carrick

**Affiliations:** 1 Department of Physiology and Aging, University of Florida College of Medicine, Gainesville, FL 32611, USA; flynnapril@ufl.edu; 2 Centre for Mental Health Research in Association with University of Cambridge, Cambridge CB2 1TN, UK; ahankir@uwo.ca; 3 Department of Neurology, Carrick Institute, Cape Canaveral, FL 32920, USA; n.alrumaihi@scfhs.org.sa; 4 School of Medicine, Cardiff University, Cardiff CF14 4YS, UK; 5 Schulich School of Medicine and Dentistry, University of Western Ontario, London, ON N6A 5C1, Canada; 6 Department of Informatics and Smart Health, Dubai Health Authority, Dubai 43111, United Arab Emirates; marad@dha.gov.ae; 7 Department of Public Health, Mohammed Bin Rashid School of Medicine, Dubai 88905, United Arab Emirates; 8 Saudi Commission for Health Specialties, Riyadh 11614, Saudi Arabia; 9 College of Medicine, University of Central Florida, Orlando, FL 32827, USA; 10 Health Professions Education, MGH Institute of Health Professions, Boston, MA 02129, USA; 11 Burnett School of Biomedical Science, University of Central Florida, Orlando, FL 32827, USA

**Keywords:** menopause, exercise, cognition, executive function, memory, neurodegeneration, BDNF, Alzheimer’s disease, neuroplasticity, postmenopausal women

## Abstract

Menopause represents a major neuroendocrine transition characterized by substantial hormonal, metabolic, vascular, and inflammatory changes that may increase vulnerability to cognitive decline and neurodegenerative disease. Declining estrogen levels during the menopausal transition influence multiple neural processes, including synaptic plasticity, cerebral glucose metabolism, mitochondrial function, neuroinflammatory signaling, and cerebrovascular regulation. Women account for nearly two-thirds of individuals diagnosed with Alzheimer’s disease, and mounting evidence suggests that menopause may represent a period of heightened neurological vulnerability. Physical exercise has emerged as one of the most promising non-pharmacological strategies for preserving cognitive health and reducing neurodegeneration risk in aging women. Current evidence demonstrates that exercise interventions after menopause are associated with improvements in executive function, memory performance, attention, and global cognition. These benefits appear to result from converging biological mechanisms that include enhanced neurotrophic signaling, increased brain-derived neurotrophic factor (BDNF) expression, improved cerebrovascular function, reduced systemic inflammation, improved insulin sensitivity, enhanced metabolic regulation, and preservation of structural brain integrity. Neuroimaging studies further demonstrate exercise-associated increases in hippocampal volume, cortical thickness, functional connectivity, and cerebral blood flow. Different exercise modalities appear to produce distinct but complementary neurological benefits. Aerobic exercise is strongly associated with improved cerebrovascular function and hippocampal integrity, resistance training demonstrates favorable effects on executive function and white matter preservation, and multimodal interventions combining aerobic, resistance, balance, and cognitively engaging activities appear to produce the broadest cognitive benefits. Emerging evidence further suggests that the timing of exercise initiation relative to menopause may influence outcomes, with earlier interventions potentially conferring greater neuroprotection. This review synthesizes current evidence regarding the effects of exercise on cognitive function in postmenopausal women, with emphasis on biological mechanisms, neuroimaging findings, exercise modality, timing considerations, and implications for neurodegeneration prevention. Understanding these relationships provides a scientific rationale for positioning exercise as a foundational strategy for preserving cognitive health and reducing neurodegenerative disease burden in postmenopausal women.

## 1. Introduction

The menopausal transition represents a major neuroendocrine event in women’s lives. Menopause, defined clinically by the permanent cessation of menstruation after loss of ovarian follicular activity, is associated with systemic physiological changes affecting metabolic regulation, cardiovascular health, and brain function [1]. Increasing evidence suggests that menopause is not solely a reproductive transition but also a neurological one, as estrogen plays a central role in maintaining neural plasticity, synaptic signaling, and cerebral metabolism.

During perimenopause, ovarian hormone production becomes more variable before declining after menopause [2,3,4]. Postmenopausal women appear to face increased vulnerability to Alzheimer’s disease (AD) and related dementias [5,6,7,8,9]. Women represent nearly two-thirds of individuals living with Alzheimer’s disease, underscoring the importance of strategies that support brain health after menopause [10]. This effort focuses on preserving cognitive function, supporting neuroplasticity, and reducing modifiable risk pathways that may interact with hormonal changes.

Mild cognitive impairment (MCI) is particularly relevant to this review because it represents an intermediate clinical state between expected cognitive aging and dementia. MCI is associated with measurable decline in one or more cognitive domains while daily function is relatively preserved, and it is a common target for exercise interventions because cognitive and functional trajectories may still be modifiable. Apolipoprotein E (APOE) genotype, especially APOE-ε4 carrier status, is also important because it modifies Alzheimer’s disease risk and may influence responsiveness to lifestyle interventions, including exercise. Accordingly, the present review considers MCI and APOE status where available, while recognizing that many exercise trials in postmenopausal women did not stratify outcomes by genotype.

Estrogen is produced in all humans, including men, although ovarian production is the dominant source of circulating estrogen in reproductive-aged women. Across the female lifespan, estrogen levels are low before puberty, fluctuate cyclically during the reproductive years, vary substantially during perimenopause, and decline to low levels after menopause [11]. Although estrogen is primarily produced in the ovaries it is also synthesized in other tissues, including the liver, adipose tissue, the heart, and notably within the brain [12]. Estrogen contributes to several neuroprotective processes in the brain and is produced locally in regions such as the hippocampus, cerebellum, amygdala, and cerebral cortex [13]. It is not surprising that estrogen modulation has been found to be a major mechanism of AD in postmenopausal women [14]. The decline in estrogen during this period has been linked to changes in hippocampal synaptic density [15,16], mitochondrial function [1], cerebral glucose metabolism [17], and neuroinflammatory signaling [18]. These biological changes may create a period of increased neurological vulnerability.

Oxidative stress is thought to be one of the main mediators of AD pathogenesis. Cellular necrosis occurs when the production of reactive oxygen species exceeds the ability of endogenous antioxidant systems to neutralize them leading to cellular damage. Due to its intense oxidative metabolism, high lipid content and comparatively low defense against antioxidants, the brain is especially sensitive to oxidative stress [19,20]. Consequently, important biological molecules (e.g., enzymes, nucleic acids and structural proteins) are vulnerable to oxidative modifications. Mitochondrial dysfunction, chronic neuroinflammation, increased levels of redox-active metal ions and amyloid-beta peptides [21,22,23] all contribute to accentuating these processes. Individually, oxidative stress may be a significant contributor to AD pathogenesis by facilitating amyloid-beta protein deposition, increasing tau phosphorylation, and inducing synaptic activity impairment and neuronal injury [24,25].

Because pharmacological approaches for preventing neurodegenerative diseases remain limited, attention has increasingly turned toward lifestyle interventions capable of supporting brain health. Among these, physical exercise has emerged as one of the most promising and accessible strategies. Exercise affects numerous physiological systems simultaneously, including neurotrophic signaling pathways, cerebrovascular function, metabolic regulation, and immune modulation.

Exercise after menopause is increasingly recognized as a modifiable behavior that may promote brain health and cognitive resilience. Structured physical activity has been associated with improvements across several cognitive domains, including executive function, memory, and global cognition. For instance, resistance training interventions have been shown to positively influence planning, cognitive flexibility, and working memory [26]. Likewise, multicomponent exercise programs that integrate aerobic activity, strength training, balance, and coordination exercises have been associated with generalized improvements in cognitive performance in postmenopausal women [27]. We propose that exercise may be especially relevant during the menopausal transition, when hormonal changes and age-related physiological processes may influence vulnerability to cognitive decline.

The cognitive effects of exercise are believed to be mediated by several complementary biological mechanisms that operate on both brain structure and function. An important mechanism in this context may be the increased expression of the neurotrophic factors, especially brain-derived neurotrophic factor (BDNF). BDNF is important for promoting neuronal survival, synaptic plasticity and neurogenesis in the hippocampus, a brain structure involved in learning and memory. It has been demonstrated that BDNF up-regulation due to exercise enhances neurogenesis in the hippocampus and promotes synaptic connectivity, which is directly associated with cognitive performance enhancement [28].

Exercise may also improve cerebrovascular function. Regular exercise improves endothelial function and increases cerebral blood flow velocity, thereby promoting delivery of oxygen and metabolic substrates to neural tissue [29]. These vascular adaptations may help preserve regions vulnerable to age-related change, such as the hippocampus and frontal cortex. Enhanced cerebral perfusion may therefore support neural health and reduce vascular contributions to cognitive decline.

Beyond neurotrophic and vascular effects, exercise has anti-inflammatory actions. Systemic inflammatory markers often increase during aging and menopause and have been implicated in cognitive decline and neurodegenerative disease. Exercise may reduce circulating pro-inflammatory cytokines and promote anti-inflammatory signaling, contributing to a physiological environment that supports neuronal integrity and resilience [27].

Another significant mechanism by which exercise can promote cognitive function involves metabolic regulation. Metabolic changes are common in postmenopausal women, including decreased insulin sensitivity and other disturbances of glucose metabolism [30,31]. Exercise improves insulin sensitivity and glucose regulation, which may benefit brain energy metabolism and reduce metabolic stress on neural tissue [32]. Improvements in metabolic function may support cognition because the brain depends heavily on efficient glucose utilization during neuronal activity.

Additional evidence of the neuroprotective effects of exercise comes from structural brain changes linked to this type of physical activity. Neuroimaging studies have shown increased gray matter volume in areas implicated in cognitive control and memory, as well as lower levels of white matter hyperintensities, pathological findings associated with small-vessel disease and aging [33]. Such structural changes further imply that exercise might also enhance immediate cognitive functioning, as well as provide longer-term maintenance of brain structure over time.

Together, these findings suggest that exercise may help maintain cognitive health after menopause through multiple biological pathways. Physical activity may counteract several mechanisms associated with cognitive decline by modulating neurotrophic signaling, vascular function, inflammatory processes, metabolic regulation, and brain structure. Exercise therefore represents a practical and potentially beneficial strategy for supporting brain health in postmenopausal women.

Practical applications depend on identifying exercise types that are safe, feasible, and effective for postmenopausal women. Network meta-analyses provide different rankings of optimal exercise types, with mind–body exercise showing the highest SUCRA score in one analysis [34] in one analysis, whereas aerobic exercise ranked highest in another. Multimodal programs combining aerobic, resistance, and cognitive components may achieve broader outcomes than single-modality interventions [28]. Sedentary behavior is associated with lower cerebral blood flow [29], reduced BDNF levels [35], and cognitive decline [36], although causal relationships remain difficult to establish.

Exercise shows potential for modifying neurodegeneration-related risk pathways. Aerobic training may increase cerebral blood flow [29], resistance training may reduce cortical white matter atrophy [37], and yoga has been associated with less gray matter loss in women at risk for Alzheimer’s disease [38]. The timing of exercise initiation relative to menopause may be important, but available evidence does not support definitive claims that earlier initiation is universally superior. Intensive lifestyle interventions may have different cognitive associations by menopausal stage and APOE-ε4 genotype [39]. In a search for the optimal prescription of exercise, we found that including moderate-intensity exercise for at least 12 weeks with sessions of 45–60 min performed 3–5 times weekly [40] was best. However, exercise prescriptions should be individualized according to menopausal stage, baseline health, cognitive status, safety considerations, and genetic risk factors when known.

## 2. Materials and Methods

### 2.1. Literature Search Strategy

A comprehensive literature search was conducted using PubMed, Scopus, Web of Science, CINAHL, and the Cochrane Library. Peer-reviewed studies published in English through January 2025 were considered for inclusion. Search terms included combinations of “menopause,” “postmenopausal women,” “exercise,” “physical activity,” “aerobic exercise,” “resistance training,” “cognition,” “executive function,” “memory,” “mild cognitive impairment,” “MCI,” “Alzheimer disease,” “apolipoprotein E,” “APOE,” “APOE-ε4,” “brain-derived neurotrophic factor,” “neurofilament light,” “NfL,” “glial fibrillary acidic protein,” “GFAP,” “neurodegeneration,” “neuroplasticity,” “cerebral blood flow,” “hormone therapy,” “menopausal hormone therapy,” and “exercise intervention.” Boolean operators and Medical Subject Heading (MeSH) terms were used where applicable. Studies were excluded because of non-postmenopausal populations, absence of exercise interventions, insufficient cognitive outcome reporting, duplicate publication, or lack of relevance to neurobiological outcomes.

Randomized controlled trials, longitudinal cohort studies, intervention studies, neuroimaging investigations, animal studies with direct mechanistic relevance, and systematic reviews examining exercise-related cognitive or neurobiological outcomes in postmenopausal women were included. Studies involving aerobic exercise, resistance training, multimodal exercise, mind–body exercise, dual-task exercise, or physical activity interventions were considered eligible. Hormone therapy studies were not treated as exercise interventions, but they were reviewed when relevant to menopausal timing, estrogen-related mechanisms, cognition, Alzheimer’s disease risk, or interpretation of exercise effects.

The initial search identified 1247 potentially relevant articles. After removal of duplicates, 914 records remained for title and abstract screening. A total of 214 full-text articles were assessed for eligibility, with 165 studies ultimately included in the final synthesis. Studies were excluded because of non-postmenopausal populations, absence of exercise interventions, insufficient cognitive outcome reporting, duplicate publication, or lack of relevance to neurobiological outcomes. A PRISMA-guided review approach was used to improve methodological transparency and study selection consistency.

### 2.2. Study Selection

Randomized controlled trials, longitudinal cohort studies, intervention studies, neuroimaging investigations, and systematic reviews examining exercise-related cognitive or neurobiological outcomes in postmenopausal women were included. Studies involving aerobic exercise, resistance training, multimodal exercise, mind–body exercise, dual-task exercise, or physical activity interventions were considered eligible. Studies focusing exclusively on pharmacologic hormone therapy without an exercise component were excluded unless directly relevant to mechanistic interpretation. Animal studies were reviewed selectively to support mechanistic explanations related to neuroplasticity, neuroinflammation, oxidative stress, and cerebrovascular physiology.

### 2.3. Data Synthesis

**Figure 1:**
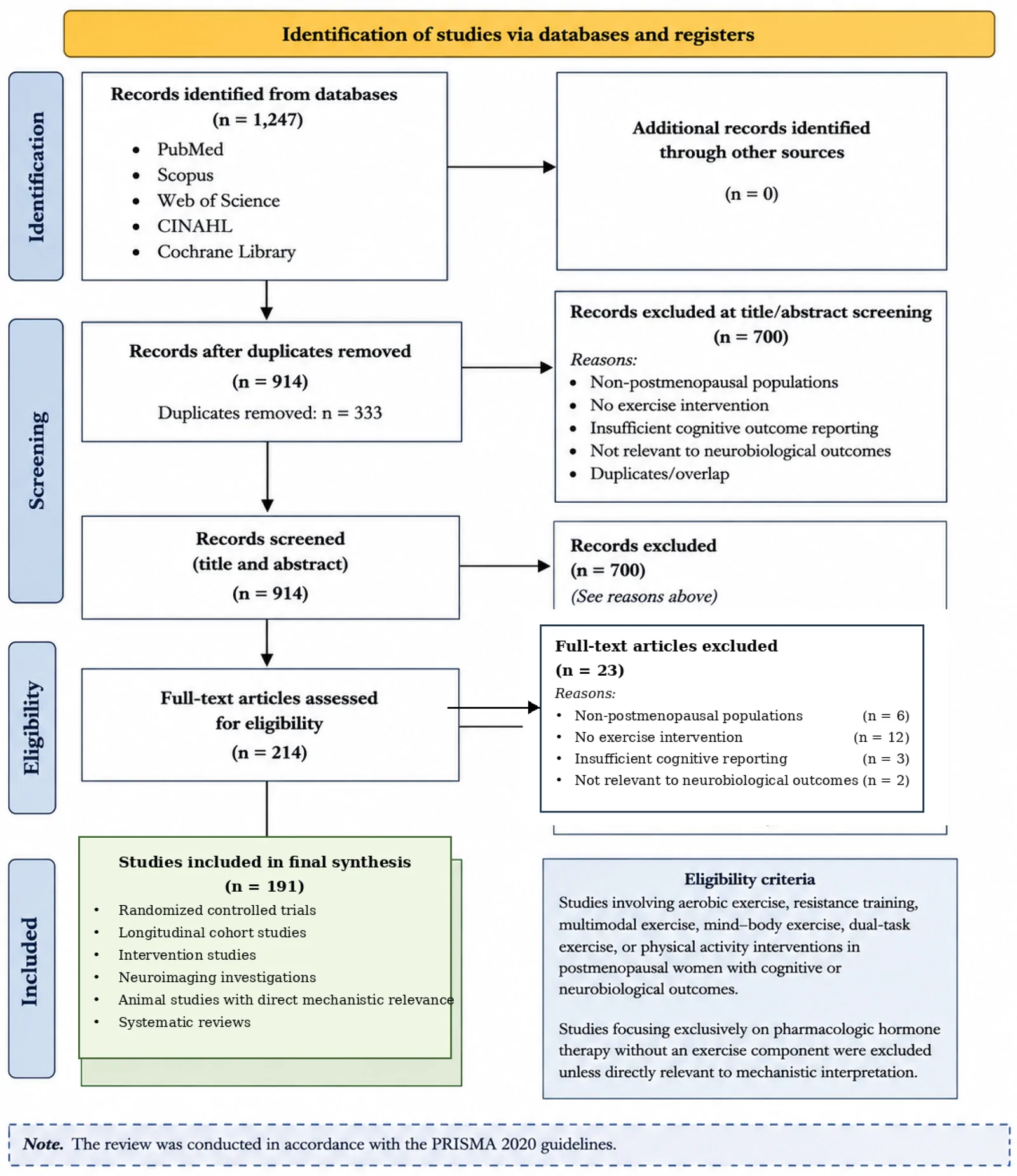
PRISMA 2020 Flow Diagram of study selection.

The literature was synthesized according to major cognitive domains and mechanistic themes, including executive function, memory performance, global cognition, neuroimaging findings, neurotrophic signaling, metabolic regulation, inflammatory pathways, cerebrovascular mechanisms, and neurodegeneration-related outcomes. Exercise modality, intensity, intervention duration, and timing relative to menopause were also examined. Figure 1 describes the PRISMA Flow Diagram of the study selection.

## 3. Results

### 3.1. Effects of Exercise on Cognitive Outcomes

#### Executive Function and Attention

A growing body of research suggests that exercise interventions can lead to meaningful improvements in executive function among postmenopausal women [27,41]. Executive function, including cognitive flexibility, working memory, attentional control, and goal-directed decision-making are particularly sensitive to age-related and hormonal changes that occur during and after the menopausal transition. Structured exercise programs, including resistance training, aerobic activity, and multicomponent interventions, have consistently been associated with enhanced performance on tasks requiring executive control. For instance, both moderate-intensity aerobic exercise and combined aerobic plus whole-body vibration interventions have demonstrated significant improvements in executive function in postmenopausal women [42]. Short-term multicomponent exercise training also improves executive function [27].

These cognitive gains are thought to arise from exercise-induced neurobiological adaptations, including increased neurotrophic signaling, improved cerebral perfusion, and enhanced metabolic regulation within frontal brain networks [43,44,45,46,47,48,49,50,51]. Collectively, these findings indicate that regular physical activity may help preserve or restore executive functioning in postmenopausal women, supporting both cognitive health and the maintenance of independence in later life.

Resistance training has also demonstrated executive-function benefits in older women. Once-weekly and twice-weekly protocols have been associated with better executive function than balance-and-toning control conditions at a 2-year follow-up [37]. In addition, high-intensity aerobic exercise in adults with mild cognitive impairment has shown sex-specific cognitive effects, with women demonstrating improvement on several executive-function measures in one trial [32]. These findings are promising but should be interpreted cautiously because they derive from a limited number of studies and may not generalize to all postmenopausal women or to all exercise prescriptions.

A multimodal exercise program is better compared to unimodal strategies, and is consistently associated with superior improvement in executive functional measures [28]. Interestingly, combined physical–cognitive exercise is associated with significant improvements in executive function better than exercise or diet alone [52]. Even a video dance intervention is associated with significant positive brain activation in areas associated with executive function [53], while a multisensory exercise program improved Montreal Cognitive Assessment scores in postmenopausal women [54].

Taken together, these findings suggest that exercise may improve cognitive function across several domains in postmenopausal women, although effect sizes, durability, and domain-specific benefits vary by intervention type, comparator group, baseline cognitive status, and study design [27,28,42,55,56,57,58,59,60].

### 3.2. Memory Performance

Memory outcomes improve across various exercise interventions. Twice-weekly resistance training promotes an increase in memory at a 2-year follow-up [54], while exercise and combined diet-exercise interventions are associated with significant improvements in memory, accompanied by increases in plasma BDNF levels and improvements in insulin sensitivity [52]. Resistance training may help maintain memory function in postmenopausal women, particularly in domains such as learning, recall, and working memory [61,62]. These benefits may reflect exercise-related physiological changes, including increased neurotrophic signaling, augmented cerebral blood flow, and improved metabolic regulation.

Resistance training may additionally induce structural and functional adaptations in key brain regions involved in memory processing, such as the hippocampus and prefrontal cortex [63]. Collectively, these results suggest that incorporating resistance training in exercise routines may be beneficial to memory performance maintenance of postmenopausal women or perhaps even improvement of cognitive function with low-cost and highly accessible strategies within the aging framework. Further, progressive resistance training also attenuates decline in memory performance in individuals with mild cognitive impairment, mediated by enhanced functional connectivity between the hippocampus and superior frontal cortex [33]. Adding to this reality, it has been observed that moderate-intensity exercise intervention can reduce the risk of memory problems in exercisers compared to controls [23].

Different types of exercises may be associated with different neurobiological changes. For example, a Pilates–cognitive dual-task intervention has been associated with improved immediate memory and evocation memory in postmenopausal women [64]. Even Yoga training improves memory and executive functions in older adults with mild cognitive impairment, with potential neuroprotective effects indicated by increased right hippocampal volume [38].

### 3.3. Global Cognitive Function

A large number of women experience changes in memory, attention and cognitive processing throughout the menopausal transition [65,66,67,68,69]. Although these changes are frequently subtle, they may indicate underlying changes to the neural physiology instigated by decreasing estrogen levels.

Estrogens also regulate synaptic plasticity and inter-neuronal communication. In experimental settings, estrogen promotes dendritic spinogenesis in the hippocampus [70,71,72,73,74,75] and long-term potentiation (a basic mechanism of learning and memory). Estrogen also modulates multiple neurotransmitter signaling systems like cholinergic, serotonergic and dopaminergic pathways which cooperate to maintain cognitive performance.

Neuroimaging studies also show metabolic and functional alterations in brain areas critical for cognition after menopause. Reduced glucose metabolism and altered functional connectivity have been identified in Alzheimer’s disease susceptible brain regions like the hippocampus, posterior cingulate cortex, and prefrontal cortex. These results point to menopause as a period of neurological vulnerability and one in which interventions that promote brain health might confer considerable long-term benefits.

Exercise interventions have improved global cognitive function across multiple studies [76]. For example, multicomponent exercise intervention showed significant improvement in Mini-Mental State Examination scores [77], while progressive resistance training significantly improved global in older adults with mild cognitive impairment [78]. Another study addressing the use of a multicomponent exercise improved Trail Making Test Part A performance and reduced physiological markers of fall risk in older women with mild cognitive impairment [36]. Movement-based interventions may also support cognition: square-stepping exercises and conventional physiotherapy training were similarly effective in improving cognition measured by the Montreal Cognitive Assessment [79]. In addition, participation in calisthenics was associated with reduced risk of cognitive decline in community-dwelling older Japanese women [57].

### 3.4. Neuroimaging Findings

Neuroimaging studies provide information about brain structure and function after exercise in postmenopausal women. Magnetic resonance imaging (MRI) and related techniques show that regular physical activity is associated with measurable changes in brain integrity, particularly in regions involved in memory, executive function, and cognitive control [80,81,82,83,84,85]. Exercise interventions have been associated with increased gray matter volume in brain regions particularly susceptible to aging and neurodegeneration, like the hippocampus and prefrontal cortex [86,87,88,89]. Some studies have also recorded reductions in white matter hyperintensities, indicators of small-vessel disease and age-related brain pathology, suggesting enhanced cerebrovascular health [90,91,92]. Functional imaging findings further suggest that exercise may influence neural connectivity and efficiency in cognitive networks [93]. Collectively, these findings suggest that physical activity can affect brain structure and function after the menopausal transition, potentially supporting neural integrity and cognitive resilience with aging.

Structural and functional brain changes have been documented across several studies. In postmenopausal women with breast cancer, aerobic exercise altered resting-state network connectivity, including decreased post-intervention connectivity between the medial prefrontal cortex and left angular gyrus [94]. Further it is important to realize that stronger between-network functional connectivity was associated with higher cardiorespiratory fitness levels [94]. In the same study context, higher cardiorespiratory fitness was associated with stronger between-network functional connectivity [94]. These findings are not necessarily contradictory as a post-intervention decrease in connectivity between specific regions may reflect improved neural efficiency or normalization of an initially maladaptive pattern, whereas greater fitness-related between-network connectivity may reflect broader network integration. Because the clinical meaning of connectivity increases or decreases depends on the network, task state, disease context, and baseline connectivity, these findings should be interpreted cautiously.

The amount of exercise necessary to evoke change in brain white matter is important for practical applications. Twice-weekly resistance training reduced cortical white matter atrophy [37], while progressive resistance training expanded gray matter in the posterior cingulate and reversed the progression of white matter hyperintensities, a biomarker of cerebrovascular disease [33]. Exercise intensity may also matter: greater average exercise intensity has been associated with greater post-intervention cortical volume, mean cortical thickness, precentral gyrus thickness, and superior parietal thickness [95].

For women with restrictions that limit conventional exercise, alternative movement-based interventions may be relevant. Yoga prevented gray matter atrophy compared with memory enhancement training, with reductions in gray matter volume observed in several cortical regions in the control condition [38]. Aerobic exercise training has also significantly increased middle cerebral blood flow velocity and decreased cerebrovascular resistance in postmenopausal women [29]. Exercise training improves cerebral blood flow during thermoregulatory challenges, with middle cerebral artery velocity maintained higher during heating after exercise compared to control conditions [96]. These vascular adaptations may occur over relatively short intervention periods; for example, basal middle cerebral artery velocity increased significantly following 16 weeks of exercise training [96].

### 3.5. Neurotrophic Factor Modulation

Brain-derived neurotrophic factor (BDNF) is a key molecular mediator linking physical exercise to brain health and cognition [97]. BDNF supports neuronal survival, synaptic plasticity, and neurogenesis, especially in hippocampal circuits involved in learning and memory [76,98,99,100,101,102,103,104,105]. Declining estrogen levels in postmenopausal women may reduce neurotrophic support and increase susceptibility to cognitive decline. Exercise may partially counter this vulnerability by increasing BDNF signaling and strengthening synaptic plasticity. Increases in BDNF related to regular physical activity have been linked to improved memory, executive function, and learning capacity, although the direction and magnitude of BDNF responses vary across populations, intervention intensity, sampling time, and assay methods.

BDNF has emerged as one plausible mechanism linking exercise to cognitive benefits. Increased daily physical activity has elevated serum BDNF concentrations in postmenopausal women and has been associated with lower memory impairment and depression risk [35]. Multimodal exercise programs have also increased plasma BDNF levels in older women, suggesting that neurotrophic signaling may contribute to cognitive improvements [28].

Exercise training increased BDNF levels in young-old women (65–74 years) [106] and aerobic conditioning has been associated with increased BDNF in some studies [107]. Regular physical activity has also been linked to increases in BDNF, nerve growth factor (NGF), and cathepsin B in postmenopausal women [108]. However, the biomarker literature remains heterogeneous, and individual studies differ in exercise dose, biological sampling, menopausal stage, and baseline health status.

Lifestyle and dietary combinations may influence BDNF levels in postmenopausal women. Exercise combined with dietary intervention improved plasma BDNF levels and insulin sensitivity, outcomes linked to memory enhancement in one trial [52]. Enjoyable or game-based exercise formats may also have clinical relevance; both exergaming and conventional therapy increased BDNF levels in elderly women, and these changes were associated with improved cognitive performance [109]. However, high-intensity aerobic exercise reduced fasting plasma BDNF levels in women with mild cognitive impairment in one study, suggesting complex regulatory mechanisms that may differ across populations and sampling conditions [32]. Overall, exercise may influence BDNF signaling and contribute to neural plasticity, synaptic remodeling, emotional regulation, and cognitive resilience [110].

### 3.6. Metabolic and Hormonal Pathways

The cognitive benefits of exercise, especially in postmenopausal women, is mediated by metabolic and hormonal pathways [111]. Key endocrine and metabolic changes associated with menopause include declining circulating estrogen, altered glucose metabolism, and increased insulin resistance. These changes may impair neuronal energy homeostasis and contribute to cognitive decline. Exercise counteracts some of these effects through improved systemic metabolic function, enhanced insulin sensitivity, and better glucose utilization [112,113,114,115,116]. Exercise may also influence hormonal signaling cascades relevant to neural health through modulation of insulin-like growth factor-1 (IGF-1), cortisol pathways, and residual estrogen signaling [117,118,119,120,121,122,123]. Collectively, these adaptations may improve cerebral energy availability, reduce metabolic stress on neurons, and support synaptic efficiency.

Metabolic improvements after exercise have mediated cognitive benefits in several studies. Exercise can increase insulin-like growth factor 1 (IGF-1), norepinephrine, osteocalcin, and uncarboxylated osteocalcin [42], and these factors have been implicated in myelination, neurogenesis, and metabolic signaling [37]. Aerobic exercise improves glucose disposal and reduced fasting plasma insulin levels in women with mild cognitive impairment [32]. Mechanistically, exercise improves insulin action through repeated skeletal-muscle contraction, activation of AMPK and related kinases, increased GLUT4 translocation, enhanced mitochondrial oxidative capacity, improved body composition, and reduced inflammatory signaling. These adaptations can improve peripheral glucose handling and may support cerebral energy metabolism. A combined physical–cognitive exercise and diet intervention increased plasma adiponectin levels and improved insulin sensitivity, but such findings should be interpreted as functional and metabolic effects unless accompanied by direct evidence of neuroprotection from neuroimaging, histology, or disease-progression outcomes [52]. Dietary changes in lifestyle in concert with exercise may be more beneficial than exercise or diet alone. For instance, aerobic exercise combined with the MIND diet results in significant changes in serum sex hormones, with improvements in cognitive function and functional status [124]. Current evidence does not establish that exercise reliably maintains estrogen or progesterone levels after menopause; rather, exercise may interact with residual hormone signaling and metabolic pathways that influence brain health [40].

Many recent studies describe the exercise effects on neurotrophic, vascular, inflammatory, neuroendocrine, and structural biomarkers [28,32,60,106,108,125,126]. Exercise interventions have produced increases in neurotrophic factors, particularly BDNF, in several studies. Inflammatory markers have also shown favorable responses, with reductions in TNF-α and IL-6 following exercise training. Structural brain changes include hippocampal volume increases relative to controls in some studies, although effects on brain morphology may be moderated by exercise intensity rather than group assignment alone.

### 3.7. Anti-Inflammatory and Antioxidant Effects

Exercise exerts important anti-inflammatory and antioxidant effects that may help protect brain health in menopausal women. The menopausal transition is often accompanied by increases in systemic inflammation and oxidative stress, processes that have been linked to cognitive decline and the development of neurodegenerative conditions such as Alzheimer’s disease [127,128,129,130,131,132,133]. Regular physical activity can help counteract these biological changes by reducing circulating pro-inflammatory cytokines and promoting the release of anti-inflammatory mediators. At the same time, exercise enhances endogenous antioxidant defenses, increasing the activity of protective enzymes that neutralize reactive oxygen species and limit oxidative damage to neural tissue [134]. These adaptations contribute to a more balanced cellular environment that supports neuronal survival and synaptic function. Through its combined anti-inflammatory and antioxidant actions, exercise may therefore reduce biological stress on the aging brain and help preserve cognitive function in postmenopausal women.

Exercise-related modulation of inflammatory pathways may support brain health by reducing TNF-α and IL-6 concentrations [27] and by influencing cortisol regulation in menopausal women [8]. High-intensity aerobic exercise reduced fasting plasma cortisol levels in women with mild cognitive impairment, which may be relevant because elevated cortisol has been associated with cognitive decline [32]. Exercise-related increases in antioxidant capacity have also been observed, including elevations in superoxide dismutase in young-old women [106], while suppression of systemic inflammation by exercise enhances neural connectivity and neurogenesis [110]. These anti-inflammatory and antioxidant adaptations may contribute to neural resilience, although they should not be interpreted as proof of disease prevention in isolation.

### 3.8. Cerebrovascular and Structural Mechanisms

Regular physical activity improves cerebrovascular function through several complementary physiological pathways that support both vascular health and brain perfusion. Exercise enhances endothelial function by increasing the production of nitric oxide, which promotes vasodilation and improves the ability of blood vessels to regulate cerebral blood flow [132,134,135,136,137,138]. At the same time, repeated increases in cardiac output during physical activity stimulate vascular remodeling and improve arterial elasticity, allowing for more efficient circulation to the brain [132,139,140,141]. Exercise also contributes to improved metabolic and inflammatory regulation, reducing vascular risk factors such as insulin resistance, hypertension, and chronic inflammation that can impair cerebrovascular integrity. Together, these adaptations enhance the delivery of oxygen and metabolic substrates to neural tissue while supporting the clearance of metabolic byproducts. By strengthening the vascular systems that sustain brain function, regular exercise may help preserve neural health and reduce the risk of cognitive decline in aging populations [112,142].

Exercise improves cerebrovascular function through multiple pathways. For example, aerobic exercise training enhances endothelial function by increasing NO synthase expression, thereby improving cerebral blood flow [96]. Structural changes in cerebral vessels and functional changes in endothelial function have been proposed as mechanisms for improved cerebral blood flow velocity [29]. Exercise also reduces basal core temperature and improves thermoregulatory control, with earlier onset of sweating and skin blood flow responses during heat stress [96] and progressive resistance training conserved cortical thickness and reduced white matter hyperintensities [33].

### 3.9. Gut–Brain Axis

The menopausal transition is associated with physiological changes that can influence the composition and function of the gut microbiome, which in turn may affect brain health through the gut–brain axis. Emerging evidence suggests that exercise may play an important role in modulating this bidirectional communication system [143]. Regular physical activity has been shown to promote a more diverse and metabolically favorable gut microbiota, increasing the abundance of beneficial microbial species that produce short-chain fatty acids and other metabolites involved in immune regulation and neural signaling [144,145]. These microbial metabolites can influence brain function through multiple pathways, including modulation of systemic inflammation, regulation of neurotransmitter synthesis, and interactions with the vagus nerve and endocrine signaling systems. In postmenopausal women, where hormonal changes may contribute to microbiome alterations and increased inflammatory tone, exercise-induced improvements in gut microbial composition may help restore metabolic and immune balance [146,147]. Through these mechanisms, exercise may indirectly support cognitive function and neural resilience by strengthening the functional connections between the gastrointestinal system and the brain.

Interestingly, a home-based multitask exercise intervention significantly increased Akkermansia muciniphila and Faecalibacterium levels while reducing Lactobacillus abundance in postmenopausal women with type 2 diabetes [148]. Increased abundance of Akkermansia is significantly correlated with improvements in both HDL levels and cognitive function, suggesting a possible gut–brain connection mediating exercise benefits [148].

### 3.10. Epigenetic Modifications

Emerging evidence indicates that epigenetic mechanisms may contribute to the cognitive and brain health effects of exercise in menopausal women [149,150]. Epigenetic regulation affects gene activity without altering DNA sequence, usually through DNA methylation, histone modification, and non-coding RNAs [151]. These mechanisms allow lifestyle factors, including physical activity, to influence expression of genes involved in neuroplasticity, antioxidant defense, and metabolic regulation. Menopausal hormonal and metabolic changes may influence epigenetic aging and cellular stress pathways. Exercise may mitigate some of these effects through adaptive epigenetic changes that support mitochondrial function, inflammatory regulation, and neuronal resilience [152]. For example, both exergaming and conventional therapy induced histone H4 and H3 hyperacetylation in elderly women, suggesting that epigenetic mechanisms may mediate some cognitive effects of exercise [32,109].

### 3.11. Comparative Effectiveness of Exercise Types

Recent research suggests that several exercise modalities can produce cognitive or neurological benefits for menopausal women, but the evidence does not support a single universally superior prescription. Aerobic exercise, such as brisk walking, cycling, or swimming, has been linked to improved cardiorespiratory fitness, cerebrovascular function, and hippocampal-relevant outcomes [112,153,154,155]. Resistance training appears particularly relevant for executive function, attention, working memory, strength, and metabolic regulation [156,157,158]. Multicomponent exercise may provide broader benefits because it concurrently targets cardiovascular, neuromuscular, balance, and cognitive systems. Mind–body activities, such as yoga or tai chi, may contribute through stress reduction, autonomic regulation, balance, and attentional control [159]. Importantly, cognitive outcomes overlap across modalities; for example, both aerobic and resistance exercise have been associated with memory and executive-function benefits. Thus, modality-specific claims should be framed as tendencies rather than mutually exclusive effects.

Comparative findings across exercise types are mixed rather than truly controversial. Two network meta-analyses provided different rankings of exercise modalities for depression and anxiety in menopausal women: one found that mind–body exercise had the highest probability of being the most effective intervention [34], whereas another found aerobic exercise to be the highest-ranked approach for depressive symptoms, followed by multimodal movement and stretching exercises [40]. Another analysis reported more pronounced effects for mind–body exercise than aerobic exercise, with individual-based formats outperforming group-based formats [110]. These differences likely reflect variation in outcomes, comparators, intervention dose, participant characteristics, and analytic methods rather than disagreement about whether exercise is beneficial.

### 3.12. Exercise Intensity and Dose–Response Relationships

Longer sessions (60–90 min) [95] and extended intervention durations (>12 weeks) produced greater effects on depressive symptoms [110]. Greater average exercise intensity was associated with greater post-intervention cortical volume and cortical thickness and specifically higher precentral gyrus thickness after intervention [95]. Mid-term exercise interventions (12 weeks to 4 months) were associated with a significant reduction in depressive symptoms, as were long-term exercise interventions (6–12 months) [160]. It appears that the amount of exercise performed makes a difference, with twice-weekly resistance training producing more widespread positive effects on cognition and brain structure than once-weekly training [37].

### 3.13. Direct Comparisons Between Exercise Types

Combined aerobic exercise with whole-body vibration appears to be more effective than aerobic exercise alone for improving neurocognitive and bone health in postmenopausal women [42]. Interestingly, video dance showed significant positive brain activation in areas associated with executive function compared to walking [53]. And some exercise types give similar outcomes to non-exercise. For instance, both square-stepping exercises and conventional physiotherapy training were equally effective in improving cognition with no significant difference between them [79].

Many combinations of exercise and non-exercise regimes have provided interesting comparisons and applications. Progressive resistance training significantly improved global cognition and increased gray matter volume in the posterior cingulate, whereas computerized cognitive training attenuated the decline in memory performance [33]. Exercise alone showed significant improvements in reading interference tendency and resting-state theta activity, with no superior benefit from combining exercise with time-restricted eating [161].

Overall, multimodal exercise programs may achieve larger or broader effects than single-modality interventions when compared on similar cognitive, physical, or functional outcomes [28]. Whole-body vibration and high-impact exercises both improve functional mobility, health-related quality of life, and depressive symptoms, with whole-body vibration showing additional benefits for bone loss prevention in some studies [162].

### 3.14. Prevention of Cognitive Decline

Aerobic exercise training may increase cerebral blood flow and attenuate age-related cerebral hypoperfusion [29], while participation in calisthenics was associated with reduced risk of cognitive decline in community-dwelling older Japanese women [57]. Resistance training improves executive function and memory while reducing cortical white matter atrophy, supporting its potential role in maintaining cognitive function [37]. Physical activity may influence cognition and dementia-related risk pathways through mechanisms that include BDNF signaling, neuroplasticity, vascular function, metabolic regulation, and inflammatory modulation [107].

Yoga training may offer brain health benefits in women at risk for Alzheimer’s disease, with right hippocampal volume increases observed after yoga compared with memory enhancement training [57]. Enjoyable exercise formats may also be useful; video dance interventions have been associated with executive-function-related brain activation and may support cognitive engagement in postmenopausal women [53].

### 3.15. Treatment of Mild Cognitive Impairment

Aerobic exercise provides a nonpharmacologic intervention that may improve executive control processes in older adults at increased risk of cognitive decline [32]. In one study, six months of high-intensity aerobic exercise showed sex-specific cognitive benefits, with women demonstrating more pronounced improvements than men [32]. Multicomponent exercise improved attention and dual-task ability and reduced fall risk in older women with mild cognitive impairment [36]. Progressive resistance training improved global cognition, while computerized cognitive training attenuated memory decline in individuals with mild cognitive impairment [33].

Combined physical–cognitive exercise and dietary intervention improved memory and executive function in postmenopausal women with obesity, suggesting potential applications for cognitive impairment risk reduction [33]. Multisensory exercise programs improved cognitive function and may support brain plasticity in postmenopausal women [54].

### 3.16. Alzheimer’s Disease-Related Biomarkers

Aerobic exercise reduces cortisol levels and improves insulin sensitivity, both of which are linked to Alzheimer’s disease-related risk pathways [32]. Progressive resistance training can reduce white matter hyperintensity progression, a cerebrovascular marker associated with dementia risk [33]. Exercise training may reduce aging-related structural and functional brain changes by improving antioxidant defenses, neurotrophic signaling, and vascular function [106]. Biomarkers relevant to Alzheimer’s disease and related dementias include amyloid-β, phosphorylated tau, total tau, neurofilament light chain (NfL), glial fibrillary acidic protein (GFAP), inflammatory markers, BDNF, insulin-related markers, and APOE genotype. NfL may index axonal injury, GFAP may reflect astroglial activation, and BDNF may reflect neuroplasticity-related signaling; however, these markers are not interchangeable and are not uniformly measured in exercise trials involving postmenopausal women. APOE-ε4 status is particularly important because it may modify baseline risk and response to intensive lifestyle or exercise interventions. Exercise increases BDNF, which enhances neural plasticity and synaptic remodeling, and is associated with increased hippocampal volume and improved connectivity within the prefrontal-limbic circuitry [110]. Future studies should include AD-related biomarker panels and genotype-stratified analyses to determine whether exercise effects differ by biological risk profile.

### 3.17. Timing Considerations and Menopausal Stage

The timing of lifestyle intervention relative to menopause may influence cognitive outcomes, but available evidence should be interpreted carefully. In a long-term analysis of women with type 2 diabetes, menopausal stage and APOE-ε4 status modified the cognitive response to an intensive lifestyle intervention [39]. This was not an exercise-only trial; it was an intensive weight-loss lifestyle intervention that included diet, physical activity, and behavioral components. The subgroup showing potentially adverse cognitive outcomes was late postmenopausal and APOE-ε4 positive, and lower BMI or weight loss in later life may itself be associated with greater Alzheimer’s disease risk. Therefore, the findings should not be interpreted as evidence that exercise is harmful in late postmenopause. Instead, they suggest that intervention intensity, weight-loss goals, menopausal timing, metabolic status, and APOE genotype may interact in ways that require individualized clinical interpretation. Interventions during perimenopause or early postmenopause may yield stronger affective or cognitive benefits in some studies [39]. Interventions during the perimenopausal stage produce greater effects on depressive symptoms than interventions during postmenopause. More genotype-stratified and menopause-staged trials are needed to help understand the factors that modulate the response to exercise for brain protection [110].

### 3.18. Cognitive Outcomes in Non-Exercising Controls

Non-exercising or lower-activity control groups often show smaller gains than exercise groups, but absence of statistically significant improvement is not equivalent to deterioration. In some studies, maintaining baseline function may itself be clinically meaningful in aging populations. For example, control participants did not show the same improvements in brain connectivity or cognitive outcomes reported in exercise groups [27,94], and in one study Timed Up and Go dual-task performance declined in the non-exercise group while improving in the exercise group [36]. These findings support the potential value of exercise for preserving or improving function, but they should not be overstated as universal evidence of decline among all sedentary women.

Sedentary behavior is associated with adverse vascular, metabolic, inflammatory, and cognitive risk profiles, including lower cerebral blood flow in some studies [29]. However, the fact that exercise improves function does not by itself prove that sedentary behavior causes neurodegeneration. The stronger conclusion is that sedentary behavior may contribute to a risk environment that is less favorable for brain health, whereas regular physical activity may improve several modifiable pathways linked to cognitive resilience [108].

### 3.19. Neurodegeneration Risk Factors

Resistance training improves executive function and memory compared with non-exercise control conditions, suggesting that physical inactivity may be associated with less favorable cognitive trajectories [37]. Twice-weekly resistance training has also reduced cortical white matter atrophy compared with non-exercise control conditions [37]. Decreased BDNF is associated with memory impairment and depression risk, indicating one possible pathway through which low physical activity may affect cognitive and affective outcomes [35]. Postmenopausal women may have lower BDNF levels than premenopausal women, and low activity levels may contribute to persistence of unfavorable neurotrophic profiles. Excessive oxidative stress can suppress neurotrophic factors such as BDNF and may affect brain perfusion and blood–brain barrier integrity [106]. Cognitive impairment is a core symptom of depression, which is prevalent in menopausal women, and sedentary behavior may exacerbate cognitive vulnerability [84]. Active women showed better outcomes in BDNF and serotonin levels than sedentary women [110]. Active women showed better outcomes in BDNF and serotonin levels than sedentary women [35].

### 3.20. Safety Profile of Exercise After Menopause

One study reported an increase in hot-flush severity among exercisers compared with controls, but after 16 weeks of exercise training there was a reduction in hot flush frequency compared with non-exercisers [59]. Several studies examining physical–cognitive exercise, Pilates–cognitive dual-task intervention, and related programs did not report serious adverse effects [52,64]. Nevertheless, symptoms such as back pain, muscle aches, palpitations, dizziness, or fainting should not be dismissed. Exercise prescriptions for postmenopausal women should include appropriate screening, gradual progression, attention to comorbid cardiovascular, metabolic, musculoskeletal, and neurologic conditions, and referral for medical evaluation when concerning symptoms occur. Overall, exercise appears feasible and generally safe when appropriately prescribed and monitored, but safety reporting should be systematic in future trials [40,163].

### 3.21. Mechanistic Convergence

Despite heterogeneity in exercise types and outcomes, several converging mechanisms appear across human and animal studies. These include increased neurotrophic signaling, improved cerebrovascular regulation, enhanced insulin sensitivity and mitochondrial function, reduced chronic inflammation and oxidative stress, improved sleep and mood regulation, and changes in neural network efficiency. Animal studies provide direct evidence for exercise-induced neural adaptations that cannot be fully assessed in human trials, including hippocampal neurogenesis, dendritic spine remodeling, synaptic plasticity, angiogenesis, reduced neuroinflammation, improved mitochondrial function, and altered amyloid-β and tau-related pathology in disease models. Human studies complement this mechanistic evidence through cognitive testing, circulating biomarkers, vascular measures, and neuroimaging outcomes. BDNF elevation is one plausible pathway [32], but it should not be treated as the sole or definitive mechanism because some studies show BDNF reductions after exercise, possibly reflecting acute versus chronic adaptations, disease status, sampling timing, or assay differences. The most defensible interpretation is that exercise affects multiple interacting systems, and cognitive benefits likely arise from coordinated vascular, metabolic, inflammatory, neurotrophic, and structural adaptations rather than from any single biomarker [52].

## 4. Limitations

Although the present review synthesizes a broad body of contemporary evidence regarding exercise and brain health in postmenopausal women, several limitations should be considered when interpreting the findings. First, the available literature is heterogeneous with respect to study design, participant characteristics, exercise modality, intervention intensity, duration, adherence, and outcome measures. This variability limits direct comparisons across studies and precludes definitive recommendations regarding a single optimal exercise prescription.

Second, many investigations evaluated cognitive performance using different neuropsychological instruments and assessed distinct cognitive domains, making quantitative comparisons challenging. Likewise, neuroimaging protocols, circulating biomarkers, and physiological outcome measures varied considerably among studies, contributing additional methodological heterogeneity.

Third, although randomized controlled trials constitute an important proportion of the evidence, many studies included relatively small sample sizes, short intervention periods, and limited long-term follow-up. Consequently, while exercise is consistently associated with improvements in cognitive function and favorable changes in neurobiological pathways, evidence demonstrating sustained prevention of dementia or modification of neurodegenerative disease progression remains limited.

Fourth, the reviewed populations were not uniform with respect to menopausal stage, chronological age, baseline physical activity, cardiometabolic health, obesity, educational attainment, cognitive status, hormone therapy exposure, or genetic risk factors such as APOE-ε4 carriage. These variables may substantially influence responsiveness to exercise but were inconsistently reported or controlled across studies.

Another limitation relates to the mechanistic literature. Biomarkers including brain-derived neurotrophic factor (BDNF), inflammatory cytokines, insulin-related pathways, neurofilament light chain (NfL), glial fibrillary acidic protein (GFAP), and neuroimaging outcomes provide important biological insights; however, relatively few studies simultaneously measured cognitive outcomes together with comprehensive biomarker panels. Consequently, many proposed mechanisms remain biologically plausible rather than definitively established.

Although this review followed a structured literature search and PRISMA-guided study selection process, it was conducted as a narrative review rather than a formal systematic review with meta-analysis. Therefore, no formal assessment of risk of bias, publication bias, or quantitative effect-size synthesis was performed. Furthermore, only English-language, peer-reviewed publications were included, introducing the possibility of language and publication bias.

An additional consideration is that not all evidence included in this review was derived exclusively from postmenopausal women. While the primary emphasis was placed on studies involving postmenopausal populations, mechanistic investigations, neuroimaging studies, studies of mild cognitive impairment, broader aging cohorts, and selected animal models were incorporated to provide biological context. These complementary sources strengthen understanding of potential mechanisms but should not be interpreted as providing the same level of direct clinical evidence as intervention studies conducted specifically in postmenopausal women. Accordingly, conclusions regarding clinical efficacy should be weighted most heavily toward studies performed in the target population, while mechanistic and broader population findings should be viewed as supportive rather than confirmatory.

Finally, although animal studies were selectively incorporated to strengthen mechanistic understanding, caution should be exercised when extrapolating preclinical findings directly to clinical populations because important physiological differences exist between experimental models and postmenopausal women.

Although the collective evidence suggests that exercise favorably influences multiple biological pathways associated with healthy brain aging, current data do not establish that exercise definitively prevents neurodegenerative disease or consistently modifies disease progression in postmenopausal women. Likewise, while several studies suggest that intervention timing relative to menopause and APOE genotype may influence cognitive outcomes, these findings remain preliminary and are derived from relatively limited and heterogeneous evidence. Additional large, prospective, genotype-stratified studies with standardized exercise interventions and long-term follow-up are needed before definitive conclusions can be drawn regarding optimal menopausal timing, APOE-dependent responses, or disease-preventive effects.

Even though the overall body of evidence favors beneficial effects of exercise on cognitive function and brain health, the literature is characterized by important methodological heterogeneity and several studies have reported variable or null findings for selected outcomes and biomarkers. Differences in exercise modality, intensity, intervention duration, adherence, participant age, menopausal stage, baseline cognitive status, cardiometabolic health, outcome measures, biomarker assessment, and neuroimaging methodologies contribute to variability in reported results and limit direct comparison across studies. In addition, many investigations enrolled relatively small cohorts and employed short intervention periods, reducing statistical power to detect long-term effects on neurodegeneration or dementia incidence. Consequently, the present findings should be interpreted as evidence supporting the potential cognitive and neurobiological benefits of exercise rather than proof of uniform efficacy across all populations, interventions, or outcome measures. Future well-powered randomized multicenter trials employing standardized exercise protocols, harmonized cognitive assessments, biomarker panels, and extended follow-up will be important for refining clinical recommendations.

Despite these limitations, the convergence of evidence across randomized trials, longitudinal studies, neuroimaging investigations, mechanistic studies, and systematic reviews consistently supports regular physical exercise as one of the most promising non-pharmacological strategies for preserving cognitive function and promoting brain health after menopause. Future large-scale, multicenter randomized trials employing standardized exercise protocols, harmonized cognitive batteries, advanced neuroimaging, fluid biomarkers, genotype-stratified analyses, and extended longitudinal follow-up will be essential to determine the optimal exercise prescription for preventing cognitive decline and neurodegenerative disease in postmenopausal women.

## 5. Strengths of This Review

This review integrates evidence from randomized controlled trials, longitudinal cohort studies, neuroimaging investigations, mechanistic studies, and systematic reviews to provide a comprehensive overview of how exercise influences cognitive function and brain health after menopause. Unlike previous reviews that focused on individual exercise modalities or isolated mechanisms, the present review synthesizes behavioral, neurobiological, vascular, metabolic, inflammatory, and neurodegenerative evidence within a single translational framework. Particular emphasis is placed on exercise prescription, timing relative to menopause, comparative effectiveness of exercise modalities, and emerging biomarkers relevant to Alzheimer’s disease and related neurodegenerative disorders.

## 6. Conclusions

For postmenopausal women seeking brain health benefits, current evidence supports moderate-intensity exercise programs of at least 12 weeks, with sessions of approximately 45–60 min performed 3–5 times weekly, while recognizing that optimal prescriptions vary by baseline fitness, comorbidities, menopausal stage, cognitive status, and goals. These quantitative recommendations should be interpreted cautiously rather than as definitive prescription thresholds. The supporting evidence is derived from studies that differed substantially in exercise modality, participant characteristics, intervention duration, adherence, and cognitive outcome measures. Furthermore, some of the evidence informing these recommendations originated from broader populations of older adults or mixed cohorts rather than exclusively postmenopausal women. Consequently, these exercise parameters should be regarded as reasonable clinical guidance based on the available literature rather than evidence establishing a universally optimal prescription for all postmenopausal women.

Multimodal programs combining aerobic, resistance, balance, and mind–body components may provide broad benefits. Cognitive challenges during exercise, such as dual-task training, may also be useful when feasible. Women in early postmenopause and those without APOE-ε4 carrier status may be more likely to benefit from intensive lifestyle interventions in some datasets, whereas late postmenopausal women, particularly those with metabolic disease or APOE-ε4 risk, may require more cautious and individualized prescriptions. Regular physical activity may support BDNF signaling, cerebral blood flow, inflammatory regulation, insulin sensitivity, and structural brain integrity, but these pathways should be understood as interacting mechanisms rather than definitive proof of neurodegeneration prevention.

Exercise after menopause is associated with meaningful brain health benefits and may improve executive function, memory, and global cognitive function through multiple converging mechanisms. The strongest evidence supports exercise as a feasible, low-cost strategy for supporting cognitive resilience and reducing modifiable risk factors linked to neurodegenerative disease. Evidence for direct prevention of neurodegeneration remains less definitive and requires trials that include longer follow-up, neuroimaging, fluid biomarkers such as NfL and GFAP, amyloid and tau markers, and APOE-stratified analyses. Conversely, sedentary behavior is associated with less favorable vascular, metabolic, inflammatory, and neurotrophic profiles and should be minimized when possible.

Exercise prescriptions should be individualized based on menopausal stage, baseline cognitive status, cardiometabolic health, safety considerations, preferences, and genetic risk factors when known. Early adoption of regular physical activity is likely advantageous, but later-life initiation may still support function, mobility, mood, and cardiometabolic health. Caution is warranted for intensive weight-loss lifestyle interventions in late postmenopausal women at elevated Alzheimer’s disease risk, and future trials should separate the effects of exercise from weight loss, diet, and behavioral intervention components. Overall, exercise remains a practical and accessible intervention for supporting brain health in postmenopausal women when appropriately timed, prescribed, and monitored.

As exercise neuroscience increasingly converges with precision medicine, biomarker discovery, advanced neuroimaging, wearable technologies, and artificial intelligence, the opportunity exists to transform individualized exercise prescription into a cornerstone of preventive neurology for postmenopausal women.

## 7. Future Directions

Although substantial progress has been made in understanding the relationship between exercise and brain health after menopause, important questions remain regarding the optimal exercise prescription, biological mechanisms, and long-term clinical implications. Future investigations should move beyond demonstrating that exercise is beneficial toward determining which interventions work best, for whom, and through which biological mechanisms.

One of the highest priorities should be the development of standardized exercise prescriptions for postmenopausal women. Current studies vary considerably in exercise modality, intensity, frequency, duration, progression, supervision, and adherence, making direct comparisons difficult. Future randomized controlled trials should adopt harmonized reporting standards and standardized intervention protocols to facilitate replication, meta-analysis, and translation into clinical practice. Consensus recommendations defining evidence-based exercise prescriptions for different menopausal stages would substantially improve clinical implementation.

A second priority is the incorporation of precision medicine into exercise research. Emerging evidence suggests that biological responsiveness to exercise may differ according to menopausal stage, cardiometabolic health, baseline cognitive status, and genetic susceptibility. Future studies should routinely stratify participants according to APOE genotype, particularly APOE-ε4 carrier status, to determine whether genetic risk modifies the cognitive and neurobiological response to exercise. Additional consideration should be given to obesity, insulin resistance, cardiovascular disease, educational attainment, and hormonal history, all of which may influence exercise responsiveness.

The use of fluid biomarkers should become routine in future clinical trials. While BDNF has received considerable attention, future investigations should incorporate broader biomarker panels that include neurofilament light chain (NfL), glial fibrillary acidic protein (GFAP), phosphorylated tau (p-tau181 and p-tau217), amyloid-β42/40 ratios, total tau, inflammatory cytokines, metabolic biomarkers, and markers of oxidative stress. Simultaneous assessment of circulating biomarkers, cognitive outcomes, and neuroimaging measures will provide a more comprehensive understanding of the biological pathways through which exercise influences brain health.

Advances in neuroimaging also present important opportunities. Future studies should combine structural MRI with diffusion tensor imaging, resting-state and task-based functional MRI, arterial spin labeling, susceptibility-weighted imaging, and quantitative volumetric analyses to characterize exercise-induced changes in brain structure, connectivity, cerebrovascular function, and neural network efficiency. Multimodal imaging may help identify imaging biomarkers that predict responsiveness to specific exercise interventions before measurable cognitive changes occur.

Another critical need is long-term longitudinal follow-up. Most available intervention studies extend for only weeks or months, limiting understanding of whether exercise alters the trajectory of cognitive aging or neurodegenerative disease. Large multicenter randomized trials with 3- to 5-year follow-up are needed to determine whether sustained physical activity delays mild cognitive impairment, Alzheimer’s disease, or other dementias while identifying the exercise dose necessary for durable neuroprotection.

Future research should also embrace personalized exercise medicine. Rather than prescribing identical exercise programs for all women, individualized prescriptions based on biological age, menopausal stage, physical fitness, cognitive status, cardiovascular risk, musculoskeletal limitations, genetic susceptibility, and patient preferences may optimize adherence and maximize clinical benefit. Adaptive exercise strategies that evolve throughout the menopausal transition may prove more effective than fixed intervention protocols.

Rapid advances in artificial intelligence (AI) provide additional opportunities for precision exercise prescription. Machine-learning algorithms integrating demographic characteristics, cognitive performance, physiological measurements, wearable sensor data, neuroimaging findings, and biomarker profiles may enable prediction of individual responsiveness to different exercise modalities. AI-assisted decision-support systems may ultimately facilitate dynamic adjustment of exercise intensity, frequency, and progression while improving adherence and clinical outcomes.

Finally, future investigations should leverage digital biomarkers and wearable technologies to provide continuous, real-world monitoring of physical activity, sleep, heart-rate variability, gait characteristics, balance, mobility, cognitive performance, and physiological recovery. These technologies may allow objective assessment of intervention adherence while identifying subtle neurological changes that precede clinically detectable cognitive decline. Integration of wearable sensors with remote cognitive assessments and electronic health records may support scalable, personalized prevention strategies applicable to large populations of postmenopausal women.

Collectively, these research priorities represent the next phase in the evolution of exercise neuroscience. Future studies integrating standardized exercise interventions, multimodal neuroimaging, fluid biomarkers, genomics, digital health technologies, and artificial intelligence have the potential to transform exercise from a broadly recommended lifestyle intervention into a personalized, mechanism-based therapeutic strategy for preserving brain health and reducing the burden of neurodegenerative disease after menopause.

We illustrate and summarize our findings in Table 1, which summarizes the characteristics of studies examining exercise interventions and brain health outcomes in peri- and postmenopausal women [27,28,29,32,33,34,35,36,37,38,39,40,42,52,53,54,57,59,64,79,94,95,96,106,107,108,109,110,124,148,160,161,162,163,164,165,166,167].

**Figure 2:**
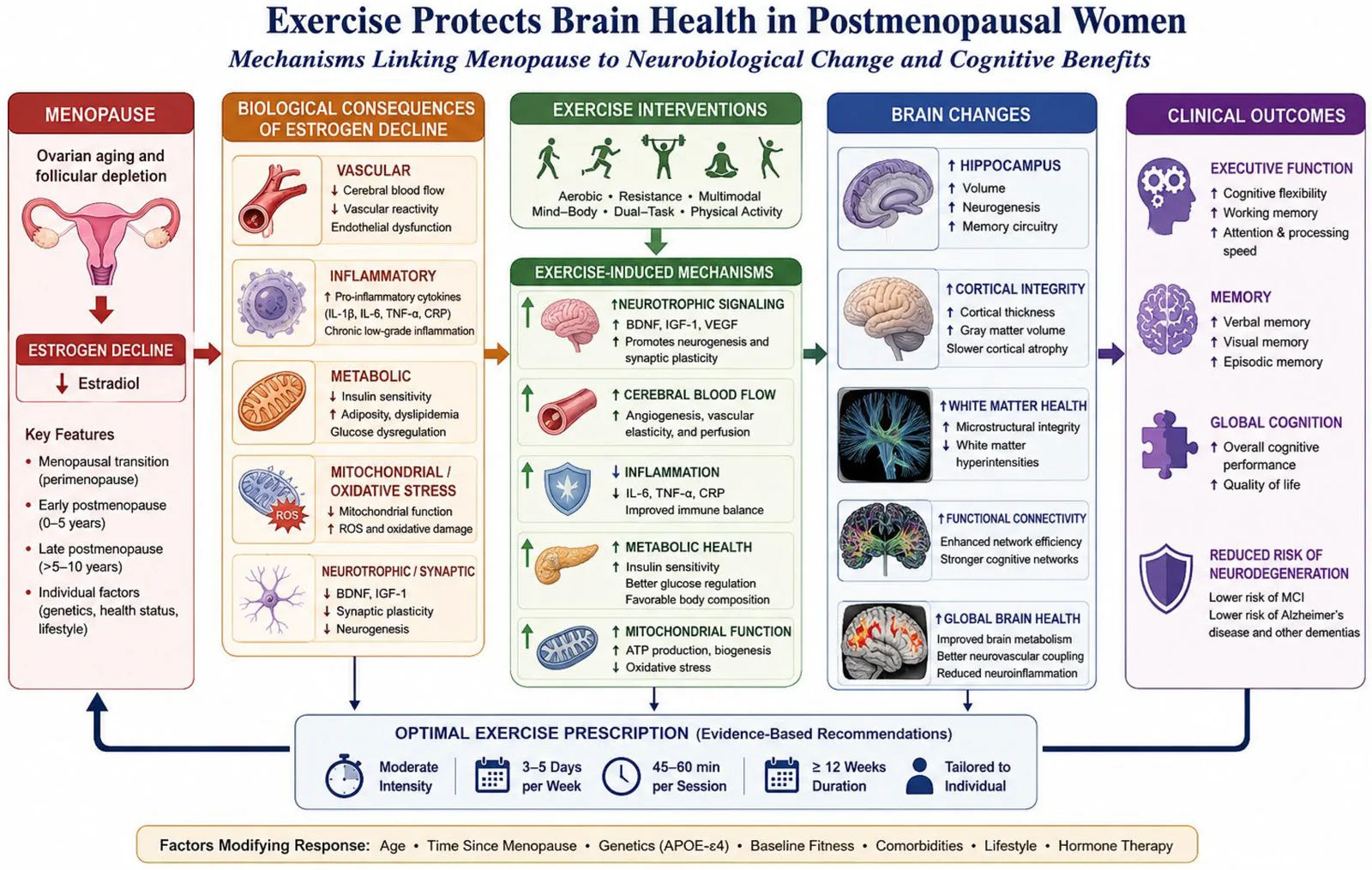
Exercise protects brain health in postmenopausal women with mechanisms linking menopause to neurobiological change and cognitive benefits.

Figure 2 is a summary cartoon generated by the authors with AI assistance describing how exercise protects brain health in postmenopausal women with mechanisms linking menopause to neurobiological change and cognitive benefits.

## Figures and Tables

**Table 1:** Characteristics of studies examining exercise interventions and brain health outcomes in peri- and postmenopausal women.

Study	Design	Population	Exercise Intervention	Duration	Brain Health Focus
Tsai et al., 2025 [42]	RCT	71 postmenopausal women	Aerobic + whole-body vibration	Not reported	Cognitive performance/neuromuscular stimulation
Akazawa et al., 2012 [29]	RCT	20 postmenopausal women; mean age 60–61 y	Aerobic cycling/walking	8 weeks	Cerebrovascular function
Ramadhana et al., 2024 [27]	RCT	30 postmenopausal women; mean age 59.8 y	Multicomponent exercise	2 weeks	Cognition/functional performance
Huang et al., 2023 [94]	RCT	34 postmenopausal breast cancer survivors	Aerobic exercise	6 months	Cognition/fatigue-related brain health
Han et al., 2024 [34]	Network meta-analysis	2170 postmenopausal women	Multiple exercise types compared	Variable	Comparative cognitive outcomes
Aiello et al., 2004 [59]	RCT	173 overweight postmenopausal women	Moderate-intensity exercise	12 months	Cognitive/health-related outcomes
Desai et al., 2022 [79]	RCT	35 postmenopausal women	Square-stepping vs. conventional exercise	Not reported	Cognitive-motor performance
Best et al., 2015 [37]	RCT	155 women aged 65–75 y	Resistance training	52 weeks	Executive function/memory
Campbell et al., 2018 [164]	RCT	Postmenopausal breast cancer survivors	Aerobic exercise	Not reported	Cognition/quality-of-life outcomes
Keawtep et al., 2024 [52]	RCT	92 postmenopausal women with obesity	Physical–cognitive exercise	3 months	Cognitive and functional outcomes
Bailey et al., 2016 [96]	Primary study	21 postmenopausal women; mean age 52 y	Aerobic exercise	16 weeks	Cerebrovascular/vascular outcomes
Bailey et al., 2016 [96]	Primary study	21 postmenopausal women	Aerobic exercise	16 weeks	Cerebrovascular/vascular outcomes
Caplan et al., 2010 [166]	Primary study	30 postmenopausal women; mean age 66 y	Aerobic weight-bearing exercise	2 years	Brain/functional aging outcomes
Silva et al., 2022 [64]	RCT	47 postmenopausal women; mean age 66 y	Pilates + cognitive dual-task	12 weeks	Dual-task cognition and function
Takahashi et al., 2019 [35]	RCT	38 postmenopausal women; mean age 70.2 y	Increased daily physical activity	8 weeks	Cognition/functional activity
Jozwiak et al., 2025 [161]	RCT	59 perimenopausal/menopausal women	Resistance + endurance circuit	12 weeks	Cognition and physical function
Roush et al., 2013 [53]	Primary study	39 postmenopausal women; mean age 55.2 y	Video dance vs. walking	Not reported	Cognitive-motor engagement
Vahed et al., 2025 [148]	RCT	70 postmenopausal women with type 2 diabetes	Home-based multitask exercise	12 weeks	Cognition/function in metabolic risk
Abd El-Hay et al., 2021 [54]	RCT	30 postmenopausal women aged 55–65 y	Multisensory exercise	4 weeks	Balance/cognitive-motor outcomes
Yassine et al., 2019 [39]	Secondary analysis of RCT	1557 women; pre/early and late postmenopause	Intensive lifestyle intervention	Long-term	Timing-dependent cognitive outcomes
Molina-Hidalgo et al., 2024 [95]	Sub-study of RCT	28 postmenopausal women; mean age 63 y	Aerobic exercise	6 months	Brain/cardiometabolic outcomes
Baker et al., 2010 [32]	RCT	33 adults with MCI; 17 women; mean age 70 y	High-intensity aerobic exercise	6 months	Cognition in MCI
Vaughan et al., 2014 [28]	RCT	49 women aged 65–75 y	Multimodal exercise	16 weeks	Global cognition
Elsayed et al., 2022 [124]	RCT	68 postmenopausal women aged 60–75 y	Aerobic exercise + MIND diet	3 months	Cognition/brain health
Perez-Lopez et al., 2017 [160]	Systematic review/meta-analysis	1943 midlife and older women	Various exercise types	Variable	Pooled cognitive/menopause-related outcomes
Wang et al., 2025 [40]	Network meta-analysis	2155 peri/postmenopausal women	Multiple exercise types compared	Variable	Comparative cognitive outcomes
Villaverde Gutierrez et al., 2012 [167]	Primary study	60 postmenopausal women aged 60–70 y	Mixed exercise with music	6 months	Cognition/quality of life
Khatib et al., 2014 [28]	RCT	49 women aged 65–75 y	Multimodal exercise	16 weeks	Cognitive performance
Sternfeld et al., 2013 [163]	RCT	248 late perimenopausal/postmenopausal women	Aerobic exercise	12 weeks	Symptoms/cognition-related outcomes
Suo et al., 2016 [33]	RCT	100 individuals with MCI; 68 females	Resistance + cognitive training	26 weeks	Cognition and brain structure/function
Yassine et al., 2020 [39]	Secondary analysis of RCT	1945 women across menopausal stages	Intensive lifestyle intervention	Long-term	Cognitive trajectories by menopausal stage
Kim et al., 2020 [108]	Primary study	52 women; 18 premenopausal, 34 postmenopausal	Resistance training	12 weeks	Cognition/biomarker-relevant outcomes
Cho et al., 2022 [106]	Primary study	24 women aged 65–84 y	Aerobic + resistance exercise	12 weeks	Cognition and physical function
Osuka et al., 2020 [57]	Prospective cohort	687 older Japanese women	Calisthenics participation	≥3 months	Longitudinal activity/cognitive-health association
Vedovelli et al., 2017 [107]	Primary study	32 institutionalized women aged 80–97 y	Multimodal strength + aerobic	3 months	Cognition/function in oldest-old women
Henrique et al., 2022 [109]	RCT	22 elderly women	Exergaming vs. conventional exercise	6 weeks	Cognition/dual-task engagement
Li et al., 2025 [110]	Meta-analysis	784 menopausal women across 16 RCTs	Multiple exercise types compared	Variable	Pooled cognitive outcomes
Sen et al., 2020 [162]	RCT	58 postmenopausal women	Whole-body vibration vs. high-impact exercise	6 months	Cognition/physical performance
Thaiyanto et al., 2020 [36]	Primary study	40 older women with MCI	Multicomponent exercise	12 weeks	Cognition in MCI
Krause-Sorio et al., 2022 [38]	RCT	22 women with subjective cognitive decline and cardiovascular risk factors	Yoga	12 weeks	Cognition/stress-related brain health

## Data Availability

All data supporting reported results can be found in the text of the paper.
